# A systematic review and meta-analysis of randomized controlled trials to reduce burden, stress, and strain in informal stroke caregivers

**DOI:** 10.1177/02692155241271047

**Published:** 2024-08-28

**Authors:** Melissa Jammal, Gregory S Kolt, Karen P Y Liu, Justin M Guagliano, Nariman Dennaoui, Emma S George

**Affiliations:** 1 School of Health Sciences, Western Sydney University, Sydney, New South Wales, Australia; 2 Department of Rehabilitation Sciences, 26680 The Hong Kong Polytechnic University, Hong Kong SAR, China; 3 Translational Health Research Institute, Western Sydney University, Sydney, New South Wales, Australia

**Keywords:** Informal caregiver, stroke, caregiver burden, strain, meta-analysis

## Abstract

**Objectives:**

To understand the nature and effectiveness of interventions aimed at improving informal stroke caregiver burden, stress, and strain.

**Data sources:**

In line with Preferred Reporting Items for Systematic Reviews and Meta-Analyses guidelines, a systematic search of CENTRAL, CINAHL, MEDLINE, Embase, APA PsycInfo, and Web of Science was conducted in May 2022.

**Review methods:**

Studies were eligible if they included an intervention designed for informal stroke caregivers, reported on caregiver burden, strain, or stress, were published in English, and used a randomized controlled trial design. An updated search was conducted in June 2024. The methodological quality of studies was appraised using the Cochrane risk-of-bias tool for randomized trials. The data were pooled, and a meta-analysis was completed for caregiver burden and strain outcomes.

**Results:**

Nineteen studies met inclusion criteria and were meta-analyzed. Interventions ranged from 4 days to 12 months. Most studies incorporated educational and/or support components. Meta-analyses revealed nonsignificant effects on caregiver burden or strain. Significant between-group differences for caregiver strain and burden were, however, found in seven studies.

**Conclusion:**

Limited studies, small sample sizes, and conflicting results made definitive conclusions on the most effective intervention characteristics for improving caregiver outcomes difficult. Of the 19 studies, seven found significant between-group differences for caregiver outcomes postintervention, and these tended to incorporate educational components and comprised between seven and nine sessions. Further high-quality research is required to identify optimal format, duration, and frequency for improving caregiver outcomes.

Globally, stroke remains the second leading cause of death, and third leading cause of death and disability combined.^1,2^ The majority of stroke survivors will live in the community following a stroke and often require ongoing assistance.^3–5^ Informal caregivers provide ongoing support to meet the needs of stroke survivors, especially with community participation, and overcoming activity restrictions.^6,7^ Providing ongoing care can result in caregiver burden, stress, and strain.^8^

Previous systematic reviews have explored the effectiveness of interventions on the health, quality of life, and/or well-being outcomes of stroke caregivers.^9–11^ A review by Legg et al.^12^ evaluated the effectiveness of interventions targeting informal stroke caregivers on outcomes such as caregiver stress and strain. This review included eight randomized controlled trials and found no significant result for stress or strain, with the exception of one study.^13^ Another review by Rubbens et al.^14^ investigated the effectiveness of interventions to reduce burden and strain in informal stroke caregivers. Findings demonstrated a dearth of evidence, and due to the variability of interventions, the optimal type and length of intervention to reduce caregiver burden and strain was unclear. The field of research has grown considerably since these reviews were published.

Therefore, the aim of this systematic review was to examine the nature and effectiveness of interventions designed to improve outcomes for informal caregivers of stroke survivors and answer the following research questions:

1. What are the characteristics of interventions designed for informal caregivers of stroke survivors?
2. What approaches were used to inform intervention development?
3. Are interventions aiming to improve caregiver burden, stress, or strain effective?

## Methods

In line with Preferred Reporting Items for Systematic Reviews and Meta-Analyses (PRISMA) guidelines (Supplementary Appendix S1),^15^ the systematic review protocol was prospectively registered on PROSPERO (ID: CRD42022315940). A systematic search of Cochrane Central Register of Controlled Trials (CENTRAL), CINAHL (EBSCO), MEDLINE (Ovid), Embase (Ovid), APA PsycInfo (EBSCO), and Web of Science was conducted in May 2022. A search strategy was developed based on terms related to population, study design, and outcomes, and date limiters were set for January 2010 through May 2022 to provide a summary of evidence based on recent published interventions in the field (Supplementary Appendix S2). No organizations, websites, or clinical registers were used to identify studies. The search strategy was reexecuted, covering the period from May 2022 to June 2024 to identify new studies.

Studies were eligible for inclusion in this review if they met the following criteria: (1) informal caregivers were aged 18 years and over, regardless of the length of the caregiving role; (2) inclusion of a nonpharmacological interventions designed for caregivers; (3) interventions were implemented in an acute, rehabilitation, or home setting; (4) caregiver burden, stress, or strain was included as an outcome; and (5) studies used a randomized controlled trial design. For the purpose of this review, informal caregivers were defined as individuals who provide unpaid care to someone who needs it within the context of an existing relationship (e.g., family member, neighbor, friend).^16^ Studies that included paid carers were excluded. Studies were also excluded if stroke survivors resided in a residential care facility as evidence suggests the needs and challenges of these carers can differ substantially.^17^

**Figure 1.**
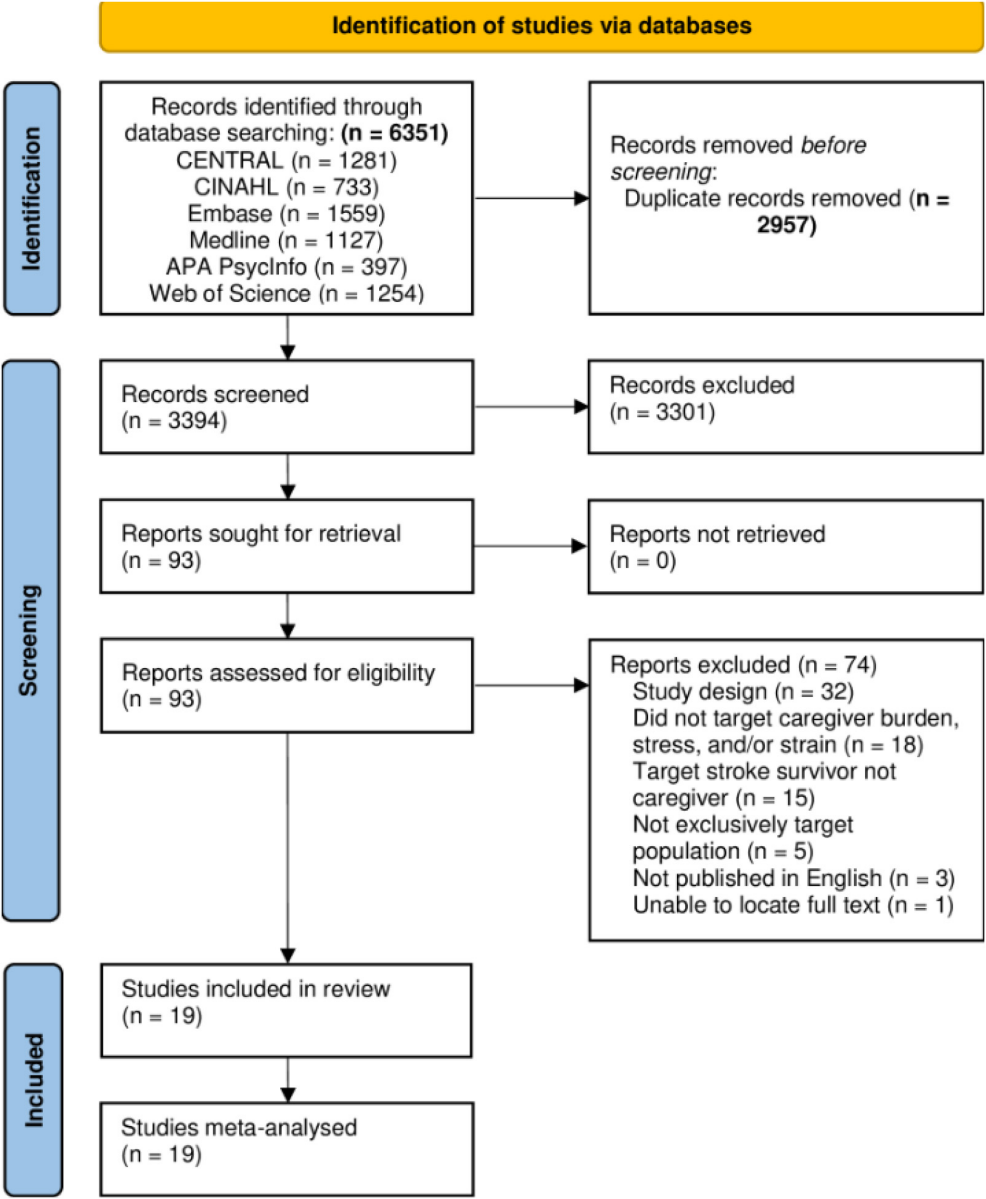
Preferred Reporting Items for Systematic Reviews and Meta-Analyses (PRISMA) flow diagram.

Following database searches, records were identified and imported into Covidence.^18^ After removal of duplicates, two reviewers (MJ and ND) independently screened the title and abstract and full text of articles based on the selection criteria. This process was monitored by a third researcher (ESG) to resolve any conflicts. Reference lists of included studies were inspected for additional studies. The full screening process is described in Figure 1. When articles were selected, two reviewers independently extracted data from each study using a standard recording form. The following data were extracted from each study: (1) author and year of publication; (2) participant characteristics and country; (3) study design, intervention details (duration, frequency, delivery, type), comparison; (4) outcome measures; and (5) main caregiver outcomes (i.e., between-group difference). The primary outcome measures extracted from each study were any changes in caregiver burden, stress, or strain. If data were missing, the corresponding author of the study was contacted by email.

The risk of bias was assessed independently by two reviewers (MJ and ESG) using the Cochrane risk-of-bias tool for randomized trials (Supplementary Figure S1).^19^ Using this tool, studies were evaluated across five domains for potential selection, performance, detection, attrition, and reporting bias. For each domain, reviewers used the tool's suggested algorithm to assign an overall rating of “low risk of bias”, “some concerns”, or “high risk of bias”, and then assigned an overall risk of bias to each study. An overall judgment of “low risk” was assigned when a study was assessed to be low risk of bias for all domains, whereas studies assigned “some concerns” for at least one domain were assigned an overall judgment of “some concerns.” A study was judged to be at “high risk of bias” where “some concerns” had been assigned for multiple domains or at least one domain was scored as “high risk.”

All studies were pooled for meta-analysis. The summary measure was the size of the intervention effect on caregiver burden and strain at intervention end. All outcomes for caregiver burden and strain were continuous and multiple measurement scales were used across the studies; thus, a standardized mean difference was used (i.e., Hedge's g).^9,20^ A random effects model was used in this study as the included studies were assumed to be varied yet similar intervention effects.^9,20^ The pooled effect was calculated for burden and strain between the intervention and control group from baseline to postintervention follow up. For each analysis, a forest plot showing the effect of the intervention on caregiver burden and strain was generated. For this study, a negative value of Hedges’ g indicates an effect favoring the intervention, while a positive value indicates the opposite. Values of Hedges’ g indicated effect sizes that were categorized as small (0.2), medium (0.5), or large (0.8 or greater). Heterogeneity across studies was assessed using the *I*^2^ statistic. In the event where heterogeneity was found (*I*^2^>50%), meta-regression was used to test the impact of potential effect modifiers (i.e., baseline caregiver age, baseline stroke survivor age, caregiver sex etc.). Stata version 17 (StataCorp LLC, College Station, TX, USA) was used to conduct the meta-analyses.

Publication bias was assessed using funnel plots (Supplementary Figure S5) and Egger's regression test.

## Results

Following database searches, 6351 records were identified. After duplicates were removed, a total of 3394 records were screened for title and abstract. A total of 3301 articles were excluded after title and abstract screening, leaving 93 full-text articles to be screened for eligibility. Two additional studies^21–23^ were identified through an updated database search in June 2024. A total of 19 studies were deemed eligible and all qualified for meta-analysis.^21–39^

Characteristics of the included studies are listed in Table 1. Of the 19 randomized controlled trials, two were feasibility studies,^30,36^ and three were pilot trials^21,32,33^ which were not powered to detect a significant change in caregiver outcomes. Of these studies, eight involved the participation of informal caregivers and stroke survivors (dyad interventions)^21–23,25,27,33,35,38^ and 11 involved caregiver participation only.^24,26,28–32,34,36,37,39^ Included studies were conducted in nine countries, China (*n* = 5), Australia (*n* = 3), Türkiye (*n* = 3), Iran (*n* = 2), United States (*n* = 2), Brazil (*n* = 1), Egypt (*n* = 1), Thailand (*n* = 1), and United Kingdom (*n* = 1). All studies assessed intervention effects directly after the intervention, four studies assessed intervention effect at six months,^29,31,35,37^ and three studies completed a 12-month follow up assessment of intervention effect.^26,35,37^

**Table 1.** Characteristics of included studies.

Study details	Intervention duration and data collection points	Study design and intervention details	Participants	Outcome measures and main findings
Ashghali-Farahani et al.^24^ Iran	Duration: 2 weeks Data collection: T0 - Baseline T1 - Post intervention (2 weeks post discharge)	Design: RCT Intervention: 8-session home care education program on breathing, relaxation, stroke education, stroke care, and anger management. 1–2 sessions at hospital, 3–6 at home within 2 weeks of discharge. Control: Routine hospital education.	Participants: 120 caregivers Intervention (*n* = 58), mean age = 43.98 ± 13.8 years, 38 female (65.5%) Control (*n* = 58), mean age = 43.41 ± 11.25 years, 45 female (77.6%) Retention rates: T1: 97% (Intervention), 97% (Control)	Caregiver burden: CBI Statistically significant between group difference (*p* < 0.001) at T1.
Bitek et al.^22^ Türkiye	Duration: 3 months Data collection: T0 – Baseline T1 - Immediately post intervention	Design: RCT Intervention: Discharge training conducted in person for approximately 40–60 min. Participants were provided training booklets including stroke education, coping with stress etc. Participants also received ongoing telephone counseling sessions once a month. Control: Routine hospital education	Participants: 69 caregivers Intervention (*n* = 34), mean age = 54.16 ± 11.73 years, 24 female (70.6%) Control (*n* = 35), mean age = 51.60 ± 14.18 years, 25 female (71.4%) Retention rates: T1: 85% (Intervention), 88% (Control)	Caregiver burden: ZBI Statistically significant between group difference (*p* < 0.012) at T1
Cheng et al.^25^ Hong Kong	Duration: 26 weeks Data collection: T0 - Baseline T1 - Post intervention (26 weeks) T2 - 1 month post intervention T3 - 3-months	Design: RCT Intervention: Strength-oriented psychoeducational program. 2 sessions before discharge, 6 biweekly sessions post discharge. Information booklet included: stroke information, caring for survivor, and caring for self. Control: Routine care	Participants: 128 caregivers Intervention (*n* = 64), mean age = 49.08 ± 12.09 years, 50 female (78.1%) Control (*n* = 64), mean age = 49.11 ± 12.90 years, 46 female (71.9%) Retention rates: T3: 86% (Intervention), 77% (Control)	Caregiver strain: CSI Statistically significantly between group difference (*p* < 0.05) at T3.
Day et al.^26^ Brazil	Duration: 30 days Data collection: T0 - Baseline T1 - Post intervention (60 days) T2 - 1 year post intervention	Design: RCT Intervention: Home care dyad intervention, 3× 1-h nurse home visits post discharge. Intervention included teaching caregiver skills (transfers, dressing), emotional support, and educational material. Control: Usual care.	Participants: 48 caregivers Intervention (*n* = 24), mean age = 53.38 ± 11.91 years, 23 female (95.8%) Control (*n* = 24), mean age = 53.54 ± 14.05 years, 19 female (79.2%) Retention rates: T1: 88% (Intervention), 96% (Control)	Caregiver burden: CBS No statistically significant difference between groups (*p* > 0.05).
Deepradit al.^23^ Thailand	Duration: 10 weeks Data collection: T0 - Baseline T1 - 4 weeks post intervention T2 - 12 weeks	Design: RCT Intervention: Family based program with 8 sessions. Sessions ranged from 15–90 min and adopted various strategies such as stress management, empowerment, time management, and home visits. Control: Usual care.	Participants: 62 caregivers Intervention (*n* = 31), mean age = 54.16 ± 15.19 years, 24 female (77.4%) Control (*n* = 31), mean age = 51.35 ± 18.09 years, 22 female (71%) Retention rates: T2: 100% (Intervention), 100% (Control)	Caregiver burden: ZBI Caregiver strain: CSI No statistically significant difference between groups in caregiver burden or strain outcomes (*p* > 0.05).
Deyhoul et al.^27^ Iran	Duration: 4 days Data collection: T0 - Baseline T1 - 2 weeks post intervention T2 – 2 months post intervention	Design: RCT Intervention: Family-centered empowerment dyad intervention with 4× 1-h sessions in hospital. Sessions included stroke prevention and treatment, problem-solving, and stroke education. Control: Usual care	Participants: 90 caregivers Intervention (*n* = 45), mean age = 41.1 ± 11 years, 33 female (73.3%) Control (*n* = 45), mean age = 40.6 ± 11.7 years, 25 female (55.6%) Retention rates: T1: 78% (Intervention), 75% (Control)	Caregiver burden: CBI Statistically significant change between groups at T1 and T2 (*p* < 0.05).
Eames et al.^28^ Australia	Duration: 3 months Data collection: T0 - Baseline T1 - 3 months	Design: RCT Intervention: Tailored information booklet and verbal reinforcement. Strategies: overcome barriers, correct misinformation, provide personalized information, reassurance and encouraging use of support networks. Control: Standard stroke care	Participants: 61 caregivers Intervention (*n* = 31), mean age = 52.97 ± 16.69 years, 21 female (67.7%) Control (*n* = 30), mean age = 58.07 ± 9.50 years, 18 female (60%) Retention rates: T1: 65% (Intervention), 73% (Control)	Caregiver strain: CSI No significant between-group differences (*p* = 0.932).
Elsheikh et al.^29^ Egypt	Duration: 6 months Data collection: T0 - Baseline T1 - 3 months T2 - 6 months	Design: RCT Intervention: 3× 120-min home visits, 6× 40-min telephone calls, and 1× 90-min peer-support session. Sessions covered stroke impact and complications, communication skills, stress management, and opportunities to share experiences. Control: Instruction booklet and standard stroke care	Participants: 110 caregivers Intervention (*n* = 55), median age = 35 years (range, 25–55), 42 female (76.4%) Control (*n* = 55), median age = 35 years (range, 25–57), 40 female (72.7%) Retention rates: T2: 91% (Intervention), 85% (Control)	Caregiver burden: ZBI - short version No statistically significant between group differences (*p* ≥ 0.05).
Fu et al.^30^ China	Duration: 9 weeks Data collection: T0: Baseline T1: One-week post intervention	Design: Feasibility RCT Intervention: Benefit finding intervention. Weekly 45-min sessions covering disease management, discovery of benefits, and positive reevaluation. Participants introduced to the “benefits discovery diary” (daily record of personal gains). Control: The content of the first 4 weeks of health education however, no strategies to promote benefit finding.	Participants: 68 caregivers Intervention (*n* = 34), mean age = 68.56 ± 5.90 years, 15 female (44.1%) Control (*n* = 34), mean age = 67.00 ± 4.42 years, 17 female (50%) Retention Rate: Not reported	Caregiver burden: ZBI Significant change between groups in caregiver burden (*p* = 0.003).
Inci et al.^31^ Türkiye	Duration: 5 weeks Data collection: T0: Baseline T1: 1-month post intervention T2: 6 months post intervention	Design: RCT Intervention: 10-session support program (5 education sessions and 5 social support sessions). Weekly 90-min sessions. Caregivers were assigned to groups and given written educational material. Control: Routine home care	Participants: 80 female caregivers. Intervention (*n* = 40), age 40–59 years (61.8%) Control (*n* = 40), age 40–59 years (58.33%) Retention rate: T2: 85% (Intervention), 90% (Control)	Family stress: FIRA-G Family strain: FIRA-G No statistically significant between group difference for family stress or strain.
				outcomes (*p* > 0.05).
LeLaurin et al.^32^ United States	Duration: 4-week or 8-week intervention Data collection: T0 - Baseline T1 - 1 week post intervention T2 - 19 weeks or 25 weeks after baseline	Design: Pilot RCT with four arms: 4-week intervention, 8-week intervention, 8-week attention control, and Standard care Intervention: Problem-solving intervention. Weekly 30–60-min telephone sessions tailored to caregiver's specific problems. Workbook and access to RESCUE website provided for the 4 or 8 weeks. Attention control: 20–30-min sessions including discussions of caregiving experiences, nurses provided active listening and paraphrasing but no advice. Standard Care: Usual care	Participants: 53 caregivers 4-week intervention (*n* = 13), 8-week intervention (*n* = 13) 8-week attention control (*n* = 13), Standard care (*n* = 14). Mean age = 60.3 ± 10.1 years, 49 female (92.5%) Retention rates: T1: 96% (Intervention), 93% (Control)	Caregiver burden: ZBI – short version No statistically significant results between group for caregiver burden (*p* > 0.05).
Marsden et al.^33^ Australia	Duration: 7 weeks Data collection: T0 - Baseline T1 - 1-week post-intervention T2 - 5 weeks post intervention	Design: Pilot RCT Intervention: Weekly group sessions for approximately 2 h and 30 min (1-h physical activity and a 1-h education component). Sessions covered stress and relaxation, stroke risk and warning signs, looking after oneself, and fatigue management). Control: Crossover trial	Participants: 17 caregivers Intervention (*n* = 9), mean age = 66.3 ± 10.1 years, 9 female (100%) Control (*n* = 8), mean age = 69.6 ± 11.5 years, 6 female (75%) Retention rates: T2: 89% (Intervention), 88% (Control)	Caregiver strain: CSI Study was not adequately powered to detect change in outcomes.
Mei et al.^34^ China	Duration: 8 weeks Data collection: T0 - Baseline T1 - Immediately post intervention T2 - 1-month post intervention T3 – 3-months	Design: RCT, three groups Group 1: Stroke survivor and caregiver attended intervention, Group 2: Caregiver only attend intervention. Intervention (Group 1 and 2): Weekly 45–60-min sessions. Prompts included diaries, letters, old photos, songs, and newspapers. Participants encouraged to recall topics such as memories, hobbies etc.	Participants: 75 caregivers Group 1 (*n* = 25), mean age = 69.67 ± 2.35 years, 20 female (80%) Group 2 (*n* = 22), mean age = 70.00 ± 2.00 years, 13 female (51.9%) Control (*n* = 28), mean age = 69.80 ± 5.58 years, 21 female (75%) Retention Rates: T3 84% (Group 1), 86% (Group 2),	Caregiver burden: CBI Statistically significant difference between groups (*p* < 0.001).
		Control: Routine health education	82% (Control)	
Minshall et al.^35^ Australia	Duration: 8 weeks + 1 booster session Data collection: T0 - Baseline T1 - 3 months T2 - 6 months T3 - 12 months	Design: RCT Intervention: 8× 1-h weekly sessions and an additional booster session at 3 months. Sessions included: understanding health, exploring strengths and vulnerabilities, stroke care education, and considering supports. Control: Standard stroke care	Participants: 84 caregivers Intervention (*n* = 35), mean age = 65 ± 13.4 years, 23 female (74%) Control (*n* = 29), mean age = 61 ± 14 years, 23 female (79%) Retention rates: T1: 39% (Intervention), 50% (Control)	Caregiver strain: MCSI No statistically significant difference between groups (*p* > 0.05)
Mou et al.^21^ China	Duration: 4 weeks Data collection: T0 - Baseline T1 - 4 weeks post discharge	Design: Pilot RCT Intervention: 3× structured 1-h psychoeducation sessions covering overview of stroke, carer training, and coping strategies. Participants also received 4× weekly 30-min follow-up telephone counseling sessions. Control: Routine stroke care.	Participants: 40 caregivers Intervention (*n* = 20), mean age = 45.61 ± 12.14 years, 9 female (45%) Control (*n* = 20), mean age = 48.10 ± 12.20 years, 10 female (50%) Retention rates: T1: 95% (Intervention), 85% (Control)	Caregiver burden: CBI Statistically significant difference between group difference (*p* < 0.013).
Walker et al.^36^ United Kingdom	Duration: 6 weeks Data collection: T0 - Baseline T1 - 6 months	Design: Feasibility RCT Intervention: Weekly 2-h biopsychosocial sessions with handouts. Sessions covered: stress and coping, relaxation exercise, and well-being action plan. The intervention was offered in a group or individual basis. Control: Routine care and usual services	Participants: 35 caregivers Intervention (*n* = 18), mean age = 63.33 ± 12.72 years, 10 female (56%) Control (*n* = 17), mean age = 61.88 ± 13.36 years, 16 female (94%) Retention rates: T1: 94% (Intervention), 76% (Control)	Caregiver burden: ZBI Study was not adequately powered to detect change in outcomes.
Wang et al.^37^ China	Duration: 12 months Data collection: T0 - Baseline T1 - 3 months T2 - 6 months T3 - 12 months	Design: RCT Intervention: Participants were invited twice/month to the hospital to receive health education and muscle relaxation. Sessions were approximately 90 min (30-min health education, 30-min demonstration, and 30-min practice) and involved systematically relaxing the major muscle groups of the body. Control: Stroke rehabilitation booklet	Participants: 110 caregivers Intervention (*n* = 55), mean age = 47.3 ± 10.4 years, 40 female (72.7%) Control (*n* = 55), mean age = 47.6 ± 10.2 years, 44 female (80.0%) Retention rates: T3: 82% (Intervention), 87% (Control)	Caregiver burden: ZBI No statistically significant difference between groups (*p* > 0.05).
Woodward et al.^38^ United States	Duration: 60 days Data collection: T0 - Baseline (7 days) T1 – Post intervention (90 days)	Design: RCT three groups Group 1: Usual care Group 2: SWCM program Group 3: SWCM + MISTT website Intervention: MISTT website included information on coping skills, stress management, stroke education, and resources. SWCM: Delivered by social workers through combination of phone calls and home visits. Control: Standard care and letters with stroke related information post-discharge.	Participants: 169 caregivers Group 1 (*n* = 58), mean age = 58.1 ± 15.5 years, 45 female (77.6%) Group 2 (*n* = 57), mean age = 57.5 ± 14.0 years, 42 female (73.7%) Group 3 (*n* = 54), mean age = 69.8 ± 13.8 years, 43 female (79.6%) Retention rates: Not reported	Caregiver burden: BCOS No statistically significant difference between groups in caregiver burden (*p* > 0.05).
Yilmaz et al.^39^ Türkiye	Duration: 8 weeks Data collection: T0 - Baseline T1 – Post intervention	Design: RCT Intervention: 60-min training session on PMR exercises and received a guided audio recording. Participants were asked to tighten their muscle groups and then relax according	Participants: 65 caregivers Intervention (*n* = 23), mean age = 47.43 ± 11.29 years, 21 female (91.3%) Control (*n* = 21), mean age = 3.43 ± 13.51 years, 16 female (76.2%)	Caregiver burden: CBI No statistically significant difference
	(8 weeks)	To commands provided by a CD. PMR exercises took approximately 28 min to complete for 3 days/week. Participants were contacted twice/week. Control: No intervention	Retention rates: T1: 70% (Int), 66% (Con)	between groups (*p* > 0.05)

BCOS: Bakas Caregiving Outcome Scale; CBI: Caregiver Burden Inventory; CBS: Caregiver Burden Scale; CSI: Caregiver Strain Index; FIRA-G: Family Index of Regeneratively and Adaptation – General; MISTT: Michigan Stroke Transitions Trial; MCSI: Modified Caregiver Strain Index; PMR: Progressive Muscle Relaxation; RCT: Randomized Controlled Trial; SWCM: Social Work Case Management; ZBI: Zarit Burden Interview.

The total sample size recruited across all included studies was 1440 informal caregivers, and sample sizes ranged from 17^33^ to 128^25^ caregivers. The caregivers in most studies were predominately female (mean 72%). Of the studies that reported caregiver mean age this ranged from 41.1^27^ to 70.0^34^ years (median 53.7 years) for the intervention group and 40.6 to 69.8 years (median 53.9 years) for the control group. Among the studies that reported length of caregiving, this ranged from 14.5 days^27^ to 10 years.^39^ From the studies that reported caregiver relationship (*n* = 14),^21,24–29,31–36,38^ most of the caregivers recruited were a partner (46.2%) or a child (34.5%) of the stroke survivor. In addition, several studies (*n* = 9)^21,22,24–26,28,29,31,38^ reported living arrangements of caregivers, and within these studies 81.7% of caregivers lived with the stroke survivor.

End of intervention retention within included studies ranged from 39%^35^ to 98%^34^ (median 81.5) in the intervention groups, and from 50%^35^ to 97%^24^ (median 75) in the control group.

Interventions were conducted within acute or rehabilitation settings (*n* = 6),^21,25,27,28,33,37^ community (*n* = 2),^29,36^ caregivers’ home (*n* = 5),^23,26,34,38,39^ or across multiple settings (*n* = 6).^22,24,30–32,35^ Nine interventions were delivered face-to-face,^24,26,27,30,31,33,34,36,37^ seven through a combination of telephone and face-to-face sessions,^21–23,25,28,29,39^ and three interventions offered flexible modes such as a website, skype, telephone, or emails.^32,35,38^ Fourteen studies included interventions that were delivered on an individual basis,^21,22,24–26,28,30,32,34,35,37–39^ four delivered on a group basis,^23,27,31,33^ and two provided a choice between individual or group delivery.^29,36^ Interventions were delivered by nurses (*n* = 10),^21–27,29,31,32^ researchers (*n* = 3),^36,37,39^ psychologists (*n* = 2),^34,35^ occupational therapists (*n* = 1),^28^ or multidisciplinary teams (*n* = 3).^30,33,38^

Majority of studies incorporated more than one component such as peer support, psychoeducation, and problem-solving.^21,23–25,27–29,31,33,36–39^ A total of nine studies incorporated an educational and/or support component focused on stroke risk factors, positioning and transfers, disease management, and stress management.^22–24,26,28,30,31,33,38^ One of these educational interventions also incorporated a physical activity component which comprised circuit training with strength training.^33^ Four studies delivered psychoeducational programs which focused on skill building and providing stroke education in combination with psychotherapeutic strategies (i.e., counseling).^21,25,29,35^ Within other studies (*n* = 3) participants received education and training on how to complete relaxation exercises,^36,37,39^ and two interventions included problem-solving techniques such as providing caregivers with virtual problems to solve.^27,32^ The remaining intervention utilized reminiscence therapy to encourage participants to recall memories on various topics including childhood, dreams, and hobbies.^34^

Interventions varied considerably in duration and total number of intervention sessions. Intervention duration ranged from 4 days^27^ to 12 months^37^ (median 56 days), and total number of intervention sessions offered ranged from 3^26^ to 24 sessions^37,39^ (median eight sessions). Intervention sessions within the studies also varied in length. The majority of sessions (*n* = 14) ranged from 30 to ≤90 min,^21–28,30–32,34,35,39^ whereas three studies had sessions that were between 90 min to 2 h and 30 min.^29,33,36^ One intervention involved participants engaging in muscle relaxation for approximately 28 min three times per week.^39^ Of the interventions that incorporated telephone contact, these sessions ranged from 30 to 60 min and telephone contact was made approximately three to six times throughout the intervention.^25,28,29^

Fourteen studies reported caregiver burden as an intervention outcome.^21–24,26,27,29,30,32,34,36–39^ Of these studies, eight^22,23,29,30,32,36,37,39^ used the Zarit Burden Interview^40^ to measure burden, and four^21,24,27,34^ used the Caregiver Burden Inventory.^41^ The remaining studies (*n* = 2)^26,38^ used the Caregiver Burden Scale (*n* = 1)^42^ or Bakas Caregiving Outcomes Scale (*n* = 1).^43^ Six studies reported on caregiver strain^23,25,28,31,33,35^ and utilized the Caregiver Strain Index^44^ (*n* = 5)^23,25,28,33,35^ or Family Strain Index^45^ (*n* = 1).^31^ Two studies^30,31^ measured caregiver stress using the Adult Carer Quality of Life Questionnaire (*n* = 1)^46^ or Family Stressors Index (*n* = 1).^45^

### Study quality

The methodological quality of studies is presented in Supplementary Figure S1. Eight studies were rated as “high risk”,^22,27,31,33,35,37–39^ six were rated as “some concerns”,^21,23,24,29,30,34^ and five were rated as “low risk” of bias overall.^25,26,28,32,36^ Two studies reported that participants were blinded to group allocation.^30,31^ Overall, 17 studies were not able to blind participants and researchers to intervention assignment due to the nature of the interventions. Concerns regarding performance bias were raised in two studies due to missing information and not using intention to treat analysis.^31,35^ In addition, some concerns were identified across eight studies due to outcome measurement which was mostly attributed to unblinded outcome assessors.^27,29,31,33,35,37–39^ Using Cohen's kappa,^47^ the interrater reliability between two reviewers was 0.80, indicating a high level of agreement. A sensitivity analysis of studies by risk of bias for burden and strain outcomes generally revealed that studies with a higher risk of bias showed more favorable effects compared to studies with a lower risk of bias (Supplementary Figure S2 and S3).

### Approaches used to inform intervention development

Thirteen studies utilized an approach such as a framework, model, or formative work to guide intervention components, identify session objectives, and for the development of strategies to achieve objectives.^21,23,25–32,34–36^ Twelve studies adopted a theoretical model or framework to guide their intervention approach.^21,25,27–29,31,32,34,36^ This included models such as the Health Belief Model,^28^ Biopsychosocial model,^36^ problem-solving and stress models (i.e., caregiving stress process model, problem-solving model),^25,32^ and family-centered models (i.e., Family-Centered Empowerment Model, the resiliency model of family stress).^27,31^ Theories and frameworks such as a dialogical and problematizing educational approach (*n* = 1),^26^ stress coping theory (*n* = 2),^30,32^ and a collaborative therapy framework (*n* = 1)^35^ were also used. Seven studies utilized formative work to guide intervention format, content, length, and delivery.^21,28,32,34–36,38^ Formative work comprised the use of qualitative methodology (*n* = 4) (i.e., questionnaires, focus groups, or interviews),^23,28,36,38^ the completion of systematic reviews (*n* = 2),^21,35^ and the use of an advisory panel and pilot study (*n* = 1).^32^

### Effectiveness of interventions

Fourteen studies evaluated the effects of a stroke caregiver intervention on caregiver burden.^21–24,26,27,29,30,32,34,36–39^ Findings from the meta-analysis (Supplementary Figure S4) revealed no overall significant difference in reduction in caregiver burden (Hedge's g = −0.40, 95% CI [−0.81 to 0.01], *p *= 0.05). Heterogeneity was high (*T*^2^* *= 0.54, *I*^2^* *= 89.2%) and no predictors explained this variation. A visual inspection of a funnel plot (Supplementary Figure S5) and Egger's regression test (*p *= 0.33) suggested no publication bias.

A small number of studies (*n* = 6) found statistically significant between-group differences for caregiver burden postintervention.^21,22,24,27,30,34^ Of these studies, five incorporated an intervention with an educational component^21,22,24,27,30^ and one used reminiscence therapy.^34^ The majority (*n* = 4) of these interventions^21,24,30,34^ included between seven and nine sessions, and one study included four sessions.^27^ These intervention sessions were delivered on a daily,^27^ weekly,^21,30,34^ monthly,^22^ or on a sporadic basis.^24^ Of these studies that found statistically significant between-group difference, end of intervention retention ranged from 78%^27^ to 97%^24^ in the intervention groups, and from 75%^27^ to 97%^24^ in the control groups. From the studies that found mean difference between the intervention and control group, the majority (*n* = 4) were rated as “some concerns”,^21,24,30,34^ and two studies received an overall rating of “high risk”.^22,27^

Six studies reported on the effectiveness of a stroke caregiver intervention on reducing caregiver strain.^23,25,28,31,33,35^ Five studies had the necessary data to be included in the meta-analysis (Supplementary Figure S6).^25,28,31,33,35^ Findings from the meta-analysis showed no effect on reducing caregiver strain (Hedge's g = −0.14, 95% CI [−0.45 to 0.17], *p *= 0.38). Heterogeneity was low (*T*^2^* *= 0.59, *I*^2^* *= 48.7%). Egger's regression test (*p *= 0.40) and review of funnel plot (Supplementary Figure S7) resembled no significant asymmetry between studies and suggested no bias from smaller studies. Thus, no statistically significant evidence of publication bias was evident.

Two studies tested the effectiveness of interventions on reducing caregiver stress.^30,31^ One study utilized a benefit finding intervention and found a nonsignificant (*p *= 0.0549) between-group difference for caregiver stress postintervention.^30^ The second study also found no statistically significant changes within the intervention or control group for family stressors (*p *= 0.512) or family distress (*p *= 0.924) outcomes.^31^

## Discussion

This review aimed to determine the nature and effectiveness of interventions designed to improve outcomes for informal stroke caregivers. Overall, meta-analyses revealed interventions were not effective in reducing burden or strain. In addition, we found no evidence that interventions were effective at reducing caregiver stress. A possible explanation for these findings could be the variation in the type of intervention, session frequency, and length of intervention. An alternative explanation could be the small sample size in pilot studies that were not powered to detect change.^21,30,32,33,36^ In addition, the majority of studies within this review did not use a codesign approach or complete formative work to inform intervention design and implementation. Evidence suggests, however, the use of a codesign approach is recommended to inform the development of relevant client-centered interventions and can lead to more effective interventions.^48,49^ Similarly, included studies were conducted across various settings, which made it challenging to draw meaningful comparisons of intervention effect.

A few studies within this review found significant between-group differences for caregiver burden and strain postintervention.^21,22,24,25,27,30,34^ The majority of these studies, however, were rated overall as “some concerns” for risk of bias, primarily due to inadequate or unclear concealment of allocation. Five of these studies, included between seven and nine intervention sessions with an educational component.^21,24,25,27,30^ Most interventions that incorporated an educational component, focused primarily on skills and training related to the stroke survivor. This is consistent with literature, which suggests within the first three months of the caregiving role, caregivers often report needing information and education on medication, treatment, and managing medical emergencies.^50^ When caregivers at this stage are not provided with education and information, it can result in uncertainty and anxiety. Similarly, a review by Bakas et al.^11^ found that interventions combining psychoeducation and skill building within five and nine sessions may improve caregiver outcomes.

In line with the findings of the current study, previous reviews found no statistically significant effects on caregiver outcomes.^9,11^ A meta-analysis by Cheng et al.^25^ found a small but nonsignificant effect of psychoeducational interventions on reducing caregiver burden in family caregivers. Whereas a meta-analysis by Chin et al.^9^ found a statistically significant reduction in depressive symptoms for technology-based interventions with an element of structured education. This review, however found no significant effects on other outcomes such as caregiver burden. Uniquely the current review reported on the use of approaches to inform intervention development, which may influence the effectiveness of interventions.

The majority of caregivers recruited within this review were a partner or child of the stroke survivor. This is reflective of statistics from countries within the Organisation for Economic Co-operation and Development.^51^ Additionally, most studies within this review included predominantly female caregivers. This is reflective of global demographics which indicate that most caregivers (paid and unpaid), regardless of location and income level, are women.^52^ The mean age of caregivers ranged from 40.6 to 70.0 years. This sample represents the average age of primary carers within countries such as Australia,^53^ United States,^54^ and United Kingdom.^55^ There is a shift in global demographics with an increase in prevalence of young carers (2%–8%) between 2001 and 2011,^56^ however, research indicates that young caregivers (aged ≤25 years) are often overlooked in research and policy.^57,58^ Based on limited evidence, young caregivers may experience unique challenges, including restricted peer networks, employment, and educational opportunities.^59^

Studies within this review recruited caregivers at different time points in their caregiving role. As the caregiving trajectory can be nonlinear, the needs of stroke caregivers can change over time. Predictors such as time spent caregiving, lifestyle restrictions, and number of caregiving tasks are associated with burden at different time points.^60,61^ Therefore, it is important that where possible, interventions are designed to suit the specific needs of caregivers at different time points.

There are several strengths and limitations within this review. Through use of the PRISMA guidelines this review was conducted in a rigorous manner, reducing inherent bias and error. Moreover, the use of meta-analysis and the Cochrane Risk of Bias tool further strengthened this review. The findings of this review should be interpreted with consideration given potential limitations. First, although approximately 68% of included studies were conducted in non-English speaking countries, only articles published in English were included. Similarly, grey literature was not searched. This may introduce language bias and exclude alternative perspectives published in nonmainstream sources. Second, meta-analysis indicated high levels of heterogeneity for caregiver burden, and no predictors explained this variation. In addition, analyses were limited to postintervention results due to variation in intervention length and data collection points, so the long-term effect could not be established. Third, the majority of studies within this review scored an overall quality rating of “some concerns” or “high risk,” indicating a lower research quality, and highlighting a need for high-quality interventions in this population. Finally, in line with previous reviews, most of the included studies recruited predominantly female caregivers, so the impact of these interventions on male caregivers remains unclear.

This review demonstrates the importance of equipping caregivers with necessary skills and support, to reduce caregiver burden, stress, and strain. High-quality trials are needed to facilitate recommendations regarding the optimal intervention, type, frequency, and length of sessions. Similarly, future studies should consider a codesign approach and recruit diverse samples of caregivers including male carers and young carers who are underrepresented in research and policy.

**Clinical messages**

- High-quality interventions that use a codesign approach to inform design and implementation are needed.
- Future studies should consider recruiting diverse samples of caregivers including male and young carers.

## Supplemental Material

sj-docx-1-cre-10.1177_02692155241271047 - Supplemental material for A systematic review and meta-analysis of randomized controlled trials to reduce burden, stress, and strain in informal stroke caregivers Supplemental material, sj-docx-1-cre-10.1177_02692155241271047 for A systematic review and meta-analysis of randomized controlled trials to reduce burden, stress, and strain in informal stroke caregivers by Melissa Jammal, Gregory S Kolt, Karen P Y Liu, Justin M Guagliano, Nariman Dennaoui and Emma S George in Clinical Rehabilitation

## References

[1] GBD 2019 Stroke Collaborators. Global, regional, and national burden of stroke and its risk factors, 1990–2019: a systematic analysis for the Global Burden of Disease Study 2019. Lancet Neurol 2021; 20: 795–820. 34487721 10.1016/S1474-4422(21)00252-0 PMC8443449

[2] Institute for Health Metrics and Evaluation. Findings from the global burden of disease study 2017. Seattle, WA: Institute for Health Metrics and Evaluation, 2018.

[3] American Stroke Association. Rehab therapy after a stroke, https://www.stroke.org/en/life-after-stroke/stroke-rehab/rehab-therapy-after-a-stroke#:∼:text=More%20than%2080%25%20of%20stroke,most%20of%20them%20at%20home. (2023).

[4] Mountain A Patrice Lindsay M Teasell R , et al. Canadian Stroke best practice recommendations: rehabilitation, recovery, and community participation following stroke. Part two: transitions and community participation following stroke. Int J Stroke 2020; 15: 789–806. 31983292 10.1177/1747493019897847

[5] Australian Institute of Health Welfare. Stroke and its management in Australia: an update. Canberra: AIHW, 2013.

[6] Sumathipala K Radcliffe E Sadler E , et al. Identifying the long-term needs of stroke survivors using the International Classification of Functioning, Disability and Health. Chronic Illn 2012; 8: 31–44. 22025770 10.1177/1742395311423848

[7] White JH Magin P Attia J , et al. Exploring poststroke mood changes in community-dwelling stroke survivors: a qualitative study. Arch Phys Med Rehabil 2008; 89: 1701–1707. 18760154 10.1016/j.apmr.2007.12.048

[8] Camak DJ . Addressing the burden of stroke caregivers: a literature review. J Clin Nurs 2015; 24: 2376–2382. 26095074 10.1111/jocn.12884

[9] Chin WJ Ho YLS Ramazanu S , et al. Effectiveness of technology-based interventions on psychological morbidities, quality of life for informal caregivers of stroke survivors: a systematic review and meta-analysis. J Adv Nurs 2022; 78: 947–967. 34904746 10.1111/jan.15130

[10] Cheng HY Chair SY Chau JPC . The effectiveness of psychosocial interventions for stroke family caregivers and stroke survivors: a systematic review and meta-analysis. Patient Educ Couns 2014; 95: 30–44. 24485756 10.1016/j.pec.2014.01.005

[11] Bakas T McCarthy MJ Miller EL . Systematic review of the evidence for stroke family caregiver and dyad interventions. Stroke 2022; 53: STROKEAHA121034090. 10.1161/STROKEAHA.121.034090 PMC9133104 35264010

[12] Legg LA Quinn TJ Mahmood F , et al. Non-pharmacological interventions for caregivers of stroke survivors. Cochrane Database Syst Rev 2011; 2011: 1–50. 10.1002/14651858.CD008179.pub2 PMC13508125 21975778

[13] Kalra L Evans A Perez I , et al. Training carers of stroke patients: randomised controlled trial. BMJ Br Med J 2004; 328: 1099–1101. 15130977 10.1136/bmj.328.7448.1099 PMC406319

[14] Rubbens E De Clerck L Swinnen E. Effectiveness of interventions to decrease the physical and mental burden and strain of informal caregivers of stroke patients: a systematic review. *Converging Clinical and Engineering Research on Neurorehabilitation II, Vols 1 and 2*. 2017, pp. 299–303.

[15] Page MJ McKenzie JE Bossuyt PM , et al. The PRISMA 2020 statement: an updated guideline for reporting systematic reviews. Br Med J 2021; 372: 71. 10.1136/bmj.n71 PMC8005924 33782057

[16] Australian Institute of Health and Welfare. *Informal carers*. Canberra: AIHW, 2021.

[17] Metzelthin SF Verbakel E Veenstra MY , et al. Positive and negative outcomes of informal caregiving at home and in institutionalised long-term care: a cross-sectional study. BMC Geriatr 2017; 17: 32. 29017453 10.1186/s12877-017-0620-3 PMC5635563

[18] Veritas Health Innovation. Covidence systematic review software, www.covidence.org (2023, accessed March 2023).

[19] Sterne JAC Savović J Page MJ , et al. Rob 2: a revised tool for assessing risk of bias in randomised trials. Br Med J 2019; 366: l4898. 10.1136/bmj.l4898 31462531

[20] Deeken F Rezo A Hinz M , et al. Evaluation of technology-based interventions for informal caregivers of patients with dementia-A meta-analysis of randomized controlled trials. Am J Geriatr Psychiatry 2019; 27: 426–445. 30642650 10.1016/j.jagp.2018.12.003

[21] Mou H Lam SKK Chien WT . Effects of a family-focused dyadic psychoeducational intervention for stroke survivors and their family caregivers: a pilot study. BMC Nurs 2022; 21: 64. 36544154 10.1186/s12912-022-01145-0 PMC9768401

[22] BİTek DE Erol Ö . The effect of discharge training and telephone counseling service on patients’ functional status and caregiver burden after stroke: a randomized controlled trial. Neurol Asia 2023; 28: 583–592.

[23] Deepradit S Powwattana A Lagampan S , et al. Effectiveness of a family-based program for post-stroke patients and families: a cluster randomized controlled trial. Int J Nurs Sci 2023; 10: 446–455. 38020842 10.1016/j.ijnss.2023.09.020 PMC10667323

[24] Ashghali Farahani M Najafi Ghezeljeh T Haghani S , et al. The effect of a supportive home care program on caregiver burden with stroke patients in Iran: an experimental study. BMC Health Serv Res 2021; 21: 46. 33858400 10.1186/s12913-021-06340-4 PMC8048267

[25] Cheng HY Chair SY Chau JPC . Effectiveness of a strength-oriented psychoeducation on caregiving competence, problem-solving abilities, psychosocial outcomes and physical health among family caregiver of stroke survivors: a randomised controlled trial. Int J Nurs Stud 2018; 87: 84–93. 30059815 10.1016/j.ijnurstu.2018.07.005

[26] Day CB Bierhals C Mocellin D , et al. Nursing Home Care Intervention Post Stroke (SHARE) 1 year effect on the burden of family caregivers for older adults in Brazil: a randomized controlled trial. Health Soc Care Community 2021; 29: 56–65. 32602588 10.1111/hsc.13068

[27] Deyhoul N Vasli P Rohani C , et al. The effect of family-centered empowerment program on the family caregiver burden and the activities of daily living of Iranian patients with stroke: a randomized controlled trial study. Aging Clin Exp Res 2020; 32: 1343–1352. 31473982 10.1007/s40520-019-01321-4

[28] Eames S Hoffmann T Worrall L , et al. Randomised controlled trial of an education and support package for stroke patients and their carers. BMJ Open 2013; 3: e002538. 10.1136/bmjopen-2012-002538 PMC3651972 23657469

[29] Elsheikh MA Moriyama M Rahman MM , et al. Effect of a tailored multidimensional intervention on the care burden among family caregivers of stroke survivors: a randomised controlled trial. BMJ Open 2022; 12: e049741. 10.1136/bmjopen-2021-049741 PMC8852666 35168963

[30] Fu B Mei Y lin B , et al. Effects of a benefit-finding intervention in stroke caregivers in communities. Clin Gerontol 2022; 45: 1317–1329. 32496892 10.1080/07317115.2020.1765062

[31] Inci FH Temel AB . The effect of the support program on the resilience of female family caregivers of stroke patients: randomized controlled trial. Appl Nurs Res 2016; 32: 233–240. 27969034 10.1016/j.apnr.2016.08.002

[32] LeLaurin JH Freytes IM Findley KE , et al. Feasibility and acceptability of a telephone and web-based stroke caregiver intervention: a pilot randomized controlled trial of the RESCUE intervention. Clin Rehabil 2021; 35: 253–265. 32907399 10.1177/0269215520957004

[33] Marsden D Quinn R Pond N , et al. A multidisciplinary group programme in rural settings for community-dwelling chronic stroke survivors and their carers: a pilot randomized controlled trial. Clin Rehabil 2010; 24: 328–341. 20176772 10.1177/0269215509344268

[34] Mei Y Lin B Li Y , et al. Effects of modified 8-week reminiscence therapy on the older spouse caregivers of stroke survivors in Chinese communities: a randomized controlled trial. Int J Geriatr Psychiatry 2018; 33: 633–641. 29266450 10.1002/gps.4833

[35] Minshall C Castle DJ Thompson DR , et al. A psychosocial intervention for stroke survivors and carers: 12-month outcomes of a randomized controlled trial. Top Stroke Rehabil 2020; 27: 563–576. 32191569 10.1080/10749357.2020.1738677

[36] Walker M Cobley C Whitehead P , et al. Biopsychosocial intervention for stroke carers (BISC): results of a feasibility randomised controlled trial. Eur Stroke J 2018; 3: 24.

[37] Wang J Liu J Li L , et al. Effect of education and muscle relaxation program on anxiety, depression and care burden in caregivers of acute stroke survivors: a randomized, controlled study. Medicine 2021; 100: e24154. 10.1097/MD.0000000000024154 PMC7850736 33530205

[38] Woodward AT Fritz MC Hughes AK , et al. Effect of transitional care stroke case management interventions on caregiver outcomes: the MISTT randomized trial. Soc Work Health Care 2021; 60: 642–655. 10.1080/00981389.2021.2009958 34933665

[39] Yilmaz CK Asiret GD Cetinkaya F , et al. Effect of progressive muscle relaxation on the caregiver burden and level of depression among caregivers of older patients with a stroke: a randomized controlled trial. Jpn J Nurs Sci 2019; 16: 202–211. 30203546 10.1111/jjns.12234

[40] Zarit SH Reever KE Bach-Peterson J . Relatives of the impaired elderly: correlates of feelings of burden. Gerontologist 1980; 20: 649–655. 7203086 10.1093/geront/20.6.649

[41] Novak M Guest C . Application of a multidimensional caregiver burden inventory. Gerontologist 1989; 29: 798–803. 2516000 10.1093/geront/29.6.798

[42] Elmståhl S Malmberg B Annerstedt L . Caregiver's burden of patients 3 years after stroke assessed by a novel caregiver burden scale. Arch Phys Med Rehabil 1996; 77: 177–182. 8607743 10.1016/s0003-9993(96)90164-1

[43] Bakas T Champion V . Development and psychometric testing of the Bakas Caregiving Outcomes Scale. Nurs Res 1999; 48: 250–259. 10494909 10.1097/00006199-199909000-00005

[44] Robinson BC . Validation of a caregiver strain index. J Gerontol 1983; 38: 344–348. 6841931 10.1093/geronj/38.3.344

[45] McCubbin H . FIRA-G family index of regenerativity and adaptation-General. *Family assessment inventories for research and practice*, 1987, pp. 285–302.

[46] Elwick H Joseph S Becker S , et al. Adult Carer Quality of Life Questionnaire (AC-QoL).

[47] Cohen J . A coefficient of agreement for nominal scales. Educ Psychol Meas 1960; 20: 37–46.

[48] Chevalier JM Buckles DJ . Thinking outside the box. In: Participatory action research: theory and methods for engaged inquiry. 1st ed. Milton, UK: Taylor & Francis Group, 2019, pp.299–317.

[49] Atkinson T Brown E Jones G , et al. “I assumed it would be somebody who had a stroke that was doing this”: views of stroke survivors, caregivers, and health professionals on tailoring a relaxation and mindfulness intervention. Healthcare (Basel) 2023; 11: 20230131. 10.3390/healthcare11030399 PMC9914663 36766974

[50] Tsai PC Yip PK Tai JJ , et al. Needs of family caregivers of stroke patients: a longitudinal study of caregivers’ perspectives. Patient Prefer Adherence 2015; 9: 449–457. 25834409 10.2147/PPA.S77713 PMC4370911

[51] OECD. *Informal carers* . 2021.

[52] United Nations Department of Economic Social Affairs. *World social report 2023: Leaving no one behind in an ageing world*. United Nations, 2023.

[53] Australian Bureau of Statistics. Disability, ageing and carers, Australia: Summary of findings, 2018. Canberra, Australia: ABS, 2019.

[54] National Alliance for Caregiving and AARP. *Caregiving in the United States* 2020. Washington, DC, May 2020.

[55] Foley N Powell A Kennedy S , et al. *Informal Carers*. Report no. 07756. London, UK: House of Commons Library, 2023.

[56] Becker S . Young carers international: reflections on 25 years of research, campaigning & life. In: *In the Swedish Family Care Competence Centre, SFCCC—Nationellt kompetenscentrum anhöriga; Proceedings of the 2nd International Conference ‘Every Child has the Right to Family’* Malmo, Sweden, 2017.

[57] Hendricks BA Kavanaugh MS Bakitas MA . How far have we come? An updated scoping review of young carers in the US. Child Adolesc Soc Work J 2021; 38: 491–504. 10.1007/s10560-021-00784-7 PMC11142575 38828384

[58] Saragosa M Frew M Hahn-Goldberg S , et al. The young carers’ journey: a systematic review and meta ethnography. Int J Environ Res Public Health 2022; 19: 1–25. 10.3390/ijerph19105826 PMC9140828 35627362

[59] Kaiser S Schulze G . Between inclusion and participation: young carers who are absent from school. J Cogn Educ Psychol 2015; 14: 314–328.

[60] Graf R LeLaurin J Schmitzberger M , et al. The stroke caregiving trajectory in relation to caregiver depressive symptoms, burden, and intervention outcomes. Top Stroke Rehabil 2017; 24: 488–495. 28618848 10.1080/10749357.2017.1338371

[61] Rigby H Gubitz G Phillips S . A systematic review of caregiver burden following stroke. Int J Stroke 2009; 4: 285–292. 19689757 10.1111/j.1747-4949.2009.00289.x

